# Functional Assessment of Cerebral Artery Stenosis by Angiography-Based Quantitative Flow Ratio: A Pilot Study

**DOI:** 10.3389/fnagi.2022.813648

**Published:** 2022-02-01

**Authors:** Kangmo Huang, Weihe Yao, Juan Du, Fang Wang, Yunfei Han, Yunxiao Chang, Rui Liu, Ruidong Ye, Wusheng Zhu, Shengxian Tu, Xinfeng Liu

**Affiliations:** ^1^Department of Neurology, Affiliated Jinling Hospital, Medical School of Nanjing University, Nanjing, China; ^2^Biomedical Instrument Institute, School of Biomedical Engineering, Shanghai Jiao Tong University, Shanghai, China; ^3^Pulse Medical Imaging Technology, Shanghai, China; ^4^Stroke Center and Department of Neurology, The First Affiliated Hospital of University of Science and Technology of China (USTC), Division of Life Sciences and Medicine, University of Science and Technology of China, Hefei, China

**Keywords:** cerebral arterial stenosis, fractional flow reserve, hemodynamics, quantitative flow ratio, artificial intelligence

## Abstract

**Background:**

Increasing attention has been paid to the hemodynamic evaluation of cerebral arterial stenosis. We aimed to demonstrate the performance of angiography-based quantitative flow ratio (QFR) to assess hemodynamic alterations caused by luminal stenoses, using invasive fractional pressure ratios (FPRs) as a reference standard.

**Methods:**

Between March 2013 and December 2019, 29 patients undergoing the pressure gradient measurement of cerebral atherosclerosis were retrospectively enrolled. Wire-based FPR was defined by the arterial pressure distal to the stenotic lesion (Pd) to proximal (Pa) pressure ratios (Pd/Pa). FPR < 0.70 or FPR < 0.75 was assumed as hemodynamically significant stenosis. The new method of computing QFR from a single angiographic view, i.e., the Murray law-based QFR, was applied to the interrogated vessel. An artificial intelligence algorithm was developed to realize the automatic delineation of vascular contour.

**Results:**

Fractional pressure ratio and QFR were assessed in 38 vessels from 29 patients. Excellent correlation and agreement were observed between QFR and FPR [*r* = 0.879, *P* < 0.001; mean difference (bias): −0.006, 95% limits of agreement: −0.198 to 0.209, respectively). Intra-observer and inter-observer reliability in QFR were excellent (intra-class correlation coefficients, 0.996 and 0.973, respectively). For predicting FPR < 0.70, the area under the receiver-operating characteristic curves (AUC) of QFR was 0.946 (95% CI, 0.820 to 0.993%). The sensitivity and specificity of QFR < 0.70 for identifying FPR < 0.70 was 88.9% (95% CI, 65.3 to 98.6%) and 85.0% (95% CI, 62.1 to 96.8%). For predicting FPR < 0.75, QFR showed similar performance with an AUC equal to 0.926.

**Conclusion:**

Computational QFR from a single angiographic view achieved comparable results to the wire-based FPR. The excellent diagnostic performance and repeatability empower QFR with high feasibility in the functional assessment of cerebral arterial stenosis.

## Introduction

Large artery atherosclerosis is a leading cause of ischemic stroke, especially in Asian populations, accounting for 30–50% of ischemic strokes in Asia (Holmstedt et al., 2013; Wang et al., 2014). Pivotal clinical trials demonstrated that the overall clinical efficacy of percutaneous transluminal angioplasty and stenting for intracranial atherosclerotic stenosis (ICAS) was substantially limited due to the high rate of perioperative complications (Chimowitz et al., 2011; Derdeyn et al., 2014). However, patients with ICAS at high risk of recurrent stroke could be better candidates for percutaneous transluminal angioplasty and stenting treatment (Alexander et al., 2019, 2020). Therefore, effective risk stratification is crucial for developing the optimal treatment strategy in the early stage of stroke.

Currently, imaging risk assessment is primarily based on the severity of anatomical stenosis, rather than functional significance. However, there is frequent discordance between anatomical stenosis and functional severity (Ahmadi et al., 2016) due to complex physiological regulation and individual variation. Therefore, we need a hemodynamic parameter beyond the severity of stenosis to quantify the functional alteration caused by the vessel narrowness.

Fractional flow reserve (FFR) is the guideline-recommended standard for assessing the hemodynamic significance of coronary artery stenosis (Knuuti et al., 2019). Nevertheless, studies evaluating the functional severity of cerebral artery stenosis are still in their infancy, with scarce published data. Since Liebeskind and Feldmann (2012) first proposed the concept of fractional flow for cerebral hemodynamics, evidence for its effectiveness has been accumulating. Considering the potential risk of hyperemic stimulation in cerebral arteries, the researchers measured the invasive fractional pressure ratios (FPRs) using pressure wires without induction of hyperemia (Han et al., 2016; Miao et al., 2016). However, the clinical application of wire-based FPR has been severely hampered by the high cost and operational demand. Surrogate indicators, such as signal intensity ratio (Leng et al., 2013) from magnetic resonance angiography (MRA), CT angiography (CTA)-derived FPR (Leng et al., 2019; Liu et al., 2017), and MRA-derived FPR (Chen et al., 2020) also have the potential to reflect the hemodynamic alteration.

Quantitative flow ratio (QFR) was calculated based on combined geometrical data and hemodynamic boundary conditions derived from coronary angiography (Tu et al., 2016, 2019; Xu et al., 2017). QFR has been widely validated showing a significant diagnostic performance and prognostic value for coronary artery stenosis (Westra et al., 2018; Biscaglia et al., 2019; Tang et al., 2021), validated in the latest multicenter randomized controlled trial (Xu et al., 2021). Recently, a new method for computing QFR from a single angiographic view, based on artificial intelligence and the Murray bifurcation fractal law, exhibited high feasibility and excellent diagnostic accuracy (Tu et al., 2021).

Therefore, we aimed to demonstrate the feasibility of applying the novel QFR to assess the hemodynamic significance of cerebral artery stenosis, using invasive FPR as a reference standard.

## Materials and Methods

### Study Design and Patients

This study enrolled patients who underwent pressure gradient measurement of cerebral atherosclerosis stenosis from March 2013 to December 2019. The exclusion criteria were as follows: (a) patients with tandem extracranial and/or intracranial stenosis, (b) extremely tortuous cerebrovascular, (c) extreme foreshortening and overlap of lesion vessel in the angiographic view, and (d) patients with intracranial tumor, arteriovenous malformation or aneurysm. This study was approved by the ethics committee of Jinling Hospital, and each patient was fully informed with written informed consent.

### Invasive Measurement of Fractional Pressure Ratio

The interventional procedure and pressure gradient measurement method were described in our published study (Han et al., 2016). In brief, angiographic images were acquired at a rate of 4 or 7.5 frames/s using a biplane flat-panel system (Artis zee; Siemens Healthcare GmbH, Erlangen, Germany). Then the pressure wire (St. Jude Medical Inc., Minneapolis, MN, United States and Uppsala, Sweden) was used to measure the mean arterial pressure of the proximal artery (Pa) and the mean arterial pressure distal to the stenotic lesion (Pd) without hyperemic stimuli. FPR, a close parameter to FFR, was calculated as the Pd/Pa.

### Analysis of Quantitative Flow Ratio and Quantitative Cerebral Angiography

Angiographic images were analyzed using a prototype software (AngioPlus Core, Pulse Medical Imaging Technology, Shanghai, China) by two independent experienced analysts blinded to FPR data. The image with the lesion segment fully expanded and clearly delineated was selected as the analysis frame. The detailed computational algorithms of the QFR have been described previously (Tu et al., 2021). They can be summarized as follows: (a) Lumen contours of the target vessels were automatically delineated by artificial intelligence using a convolutional neural network of the U-Net architecture (Ronneberger, 2017). (b) Reference vessel size was reconstructed by calculating a step-down reference diameter function according to the Murray bifurcation fractal law (Murray, 1926; Tu et al., 2015). (c) After obtaining the stenosis geometry, pressure drop was calculated according to fluid dynamics equations (Kirkeeide, 1991), assuming the blood density and viscosity of 1,060 kg/m^3^ and 0.0035 kg/(m⋅s), respectively. Additionally, the QFR computations were performed based on the empiric mean flow velocities (0.40 m/s of internal carotid artery and vertebrobasilar artery, and 0.60 m/s of MCA) (Pase et al., 2012; Tegeler et al., 2013). Finally, the QFR of the target vessel and its branches and the quantitative cerebral angiography data [minimal lumen diameter, percent diameter stenosis (DS%) and percent area stenosis (AS%), etc.] were available from the software.

### Statistical Analysis

Correlation analyses were detected by Spearman’s correlation test. Bland–Altman analyses were conducted to compare the agreement of different continuous variables. Generalized additive models were used to investigate potential non-linear relationships (Verbeke, 2007). Coefficients of determination (*R*^2^) and root mean square errors (RMSE) were calculated to assess the models. To check the intra-observer and inter-observer reliabilities in the QFR analyses, repeated analyses were performed on all subjects. The agreement of the repeated data was evaluated using the intra-class correlation coefficient (ICC).

Since there is no acknowledged cut-off value of FPR in cerebral arterial stenosis, two empiric cut-offs (FPR = 0.70 and FPR = 0.75) (Petraco et al., 2013; Han et al., 2016) were set to explore the diagnostic performance of QFR and DS%. The area under the curve (AUC) of receiver-operating characteristic (ROC) curves was calculated to evaluate the predictive accuracy. The optimal cut-off values of DS% were determined by the maximum Youden index.

Statistical analysis was performed using MedCalc (version 20.0.1, MedCalc Software, Ostend, Belgium) and R statistical software [(version 4.0.3, R Core Team (2020)]. Two-sided *P-*values of less than 0.05 were considered to be statistically significant.

## Results

### Baseline Clinical and Lesion Characteristics

A total of 29 patients (mean age 59.1 ± 8.7 years, 19 men) were included in this study. There were 25 lesions located in the anterior circulation and 4 in the posterior circulation (Table 1). Among these patients, nine patients underwent percutaneous transluminal angioplasty and stenting and received FPR measurement pre- and post-stenting. Finally, the FPR data of 38 vessels from 29 patients were obtained, and the mean value of FPR was 0.66 ± 0.21. The mean values of QFR were 0.66 ± 0.23.

**TABLE 1:** Baseline information and the results of FPR and QFR.

No.	Gender	Age (year)	Lesion vessel	Status	FPR	QFR	DS%	AS%
1	M	61	ICA C7	Pre-stent	0.60	0.54	55	79
				Post-stent	0.92	0.93	34	57
2	F	41	MCA	Baseline	0.49	0.39	53	78
3	M	49	MCA	Baseline	0.78	0.77	42	67
4	M	71	MCA	Baseline	0.74	0.75	50	75
5	F	71	ICA C6	Baseline	0.74	0.80	47	72
6	M	54	ICA C4	Pre-stent	0.64	0.61	58	83
				Post-stent	0.91	0.93	29	49
7	F	56	MCA	Pre-stent	0.33	0.59	57	81
				Post-stent	0.83	0.99	12	23
8	F	55	ICA C6	Pre-stent	0.47	0.35	63	86
				Post-stent	0.56	0.74	50	75
9	M	56	VA	Baseline	0.85	0.78	48	73
10	F	65	MCA	Baseline	0.50	0.61	51	76
11	M	55	MCA	Baseline	0.86	0.82	41	65
12	M	62	ICA C4	Baseline	0.75	0.61	56	80
13	M	75	MCA	Baseline	0.85	0.76	43	67
14	F	58	MCA	Baseline	0.74	0.68	50	75
15	M	54	ICA C4	Baseline	0.39	0.33	67	89
16	F	48	VA	Pre-stent	0.53	0.43	59	83
				Post-stent	0.88	0.86	42	66
17	F	48	ICA C7	Baseline	0.60	0.60	60	84
18	M	65	ICA C7	Pre-stent	0.19	0.14	81	96
				Post-stent	0.56	0.91	30	51
19	M	47	BA	Pre-stent	0.76	0.76	50	75
				Post-stent	0.99	0.91	28	48
20	M	65	MCA	Baseline	0.59	0.58	51	76
21	M	58	ICA C6	Baseline	0.23	0.13	78	95
22	M	69	MCA	Pre-stent	0.41	0.38	62	85
				Post-stent	0.87	0.88	32	54
23	F	63	MCA	Pre-stent	0.40	0.48	55	80
				Post-stent	0.90	0.94	27	47
24	M	56	MCA	Baseline	0.36	0.32	64	87
25	M	66	ICA C3	Baseline	0.76	0.62	55	80
26	M	56	ICA C3	Baseline	0.85	0.80	54	79
27	M	77	ICA C3	Baseline	0.86	0.80	45	70
28	M	59	MCA	Baseline	0.58	0.54	54	79
29	M	54	VA	Baseline	0.91	0.87	40	64

*FPR, fractional pressure ratio; QFR, quantitative flow ratio; DS, diameter stenosis; AS, area stenosis; M, male; F, female; ICA, internal carotid artery; MCA, middle cerebral artery; VA, vertebral artery; BA, basilar artery.*

**FIGURE 1:**
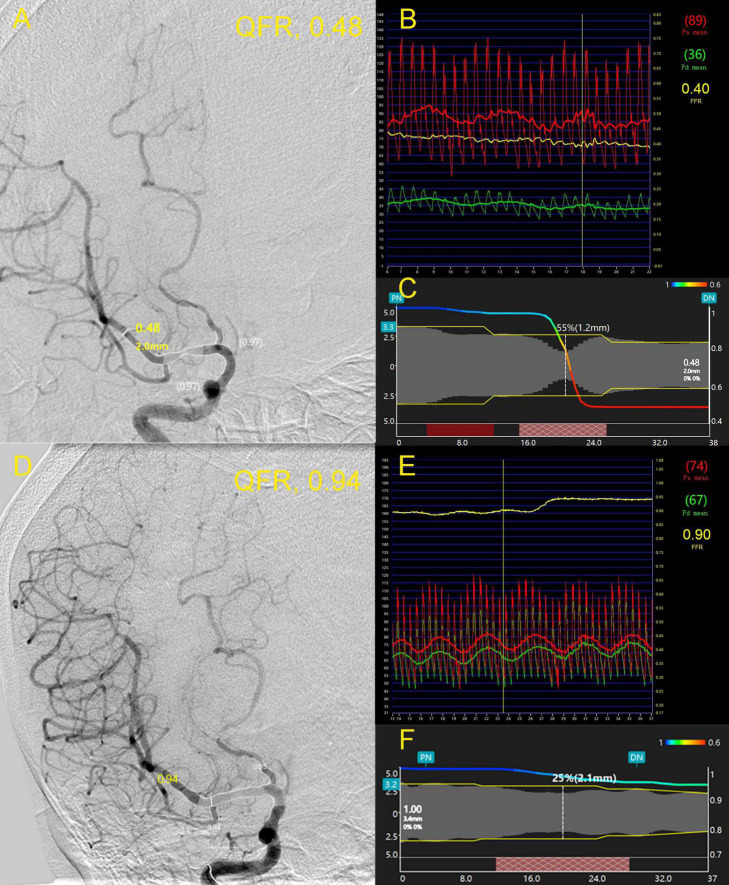
A 63-year-old female was admitted due to newly developed left limb weakness and numbness. Panel **(A)** shows an intermediate lesion (white arrow) at the right middle cerebral artery (MCA). The wire-based fractional pressure ratios (FPRs) were 0.40 **(B)** while the computed quantitative flow ratio (QFR) at the lesion was 0.48 with a remarkable pressure drop **(C)**. After stenting, the stenosis of the right MCA was relieved efficiently **(D)**. FPR increased to 0.90 **(E)** and the computed QFR was also up to 0.94 with a minor pressure drop **(F)**. FPR, fractional pressure ratio; QFR, quantitative flow ratio; MCA, middle cerebral artery.

An illustrative case was presented in Figure 1. A 63-year-old female was admitted due to newly developed left limb weakness and numbness. The MRI of the brain revealed multiple acute infarctions in the right middle cerebral artery (MCA) territory. A cerebral angiogram showed an intermediate lesion at the right MCA (Figure 1A). The wire-based FPR was 0.40 (Figure 1B) while the computed QFR at the lesion was 0.46 with a remarkable pressure drop (Figure 1C). After MCA stenting, the stenosis of the right MCA was relieved efficiently (Figure 1D). The wire-based FPR increased to 0.90 (Figure 1E) and the computed QFR was also up to 0.94 with a minor pressure drop (Figure 1F).

### Relationship Between Quantitative Flow Ratio and Fractional Pressure Ratio

**FIGURE 2:**
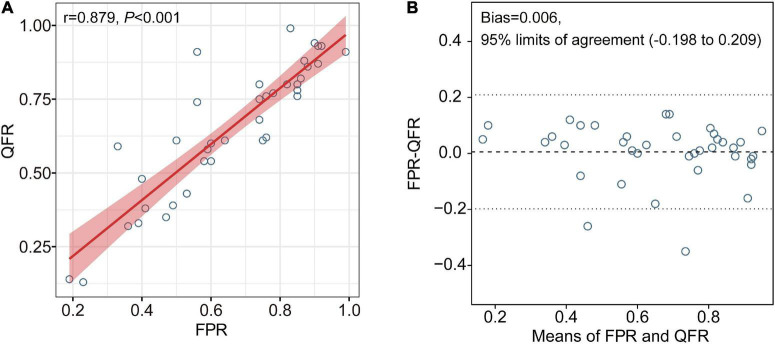
Correlation and agreement between QFR and FPR. Excellent correlation and agreement were observed between QFR and FPR (**A**,**B**, respectively). FPR, fractional pressure ratio; QFR, quantitative flow ratio.

Quantitative flow ratio showed good correlation (*r* = 0.879, *P* < 0.001) with FPR (Figure 2A). Excellent agreement was observed between QFR and FPR [mean difference (bias): 0.006, 95% limits of agreement: −0.198 to 0.209, Figure 2B). The difference between FPR and QFR showed no significant difference among different vessels (internal carotid artery, MCA, vertebral artery, and basilar artery) (*P* = 0.368). Excellent intra-observer and inter-observer reliability in QFR was confirmed, with an ICC of 0.996 and 0.973, respectively.

**FIGURE 3:**
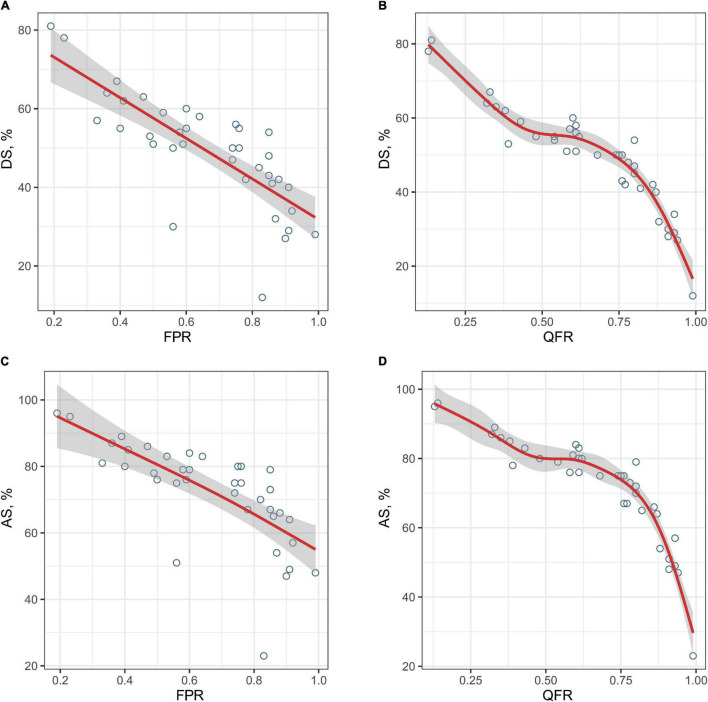
The relationships between the degree of stenosis and FPR or QFR. The fitted curves demonstrate the relationships between the degree of stenosis and FPR or QFR (**A**, DS% and FPR; **B**, DS% and QFR; **C**, AS% and FPR; and **D**, AS% and QFR). FPR, fractional pressure ratio; QFR, quantitative flow ratio; DS, diameter stenosis; AS, area stenosis.

The correction between DS% or AS% and FPR were significant (*r* = −0.815, *P* < 0.001; *r* = −0.817, *P* < 0.001, respectively), however, which were smaller than that of QFR and FPR. Figure 3 shows the relationships between DS%/AS% and FPR/QFR. To further explore the relationship between the variables, we fitted the data by linear regression models and generalized additive models, respectively. As shown in Table 2, values of RMSE and *R*^2^ between the percentage of the stenosis (DS% or AS%) and the FPR had changed considerably, indicating a non-linear relationship between them. However, these parameters between QFR and FPR change negligibly, suggesting a linear relationship.

**TABLE 2:** Statistical results of goodness-of-fit test for different regression models.

Variable 1	Variable 2	Linear regression models		Generalized additive models		RMSE different	*R*^2^ different
		RMSE	*R* ^2^	RMSE	*R* ^2^		
DS%	FPR	0.130	0.602	0.112	0.689	14%	14%
DS%	QFR	0.092	0.824	0.073	0.883	21%	7%
AS%	FPR	0.148	0.491	0.112	0.691	24%	41%
AS%	QFR	0.119	0.709	0.074	0.881	38%	24%
QFR	FPR	0.096	0.786	0.096	0.786	0%	0%

*RMSE, root mean square errors; DS, diameter stenosis; AS, area stenosis; FPR, fractional pressure ratio; QFR, quantitative flow ratio.*

### Diagnostic Performance of Quantitative Flow Ratio

**FIGURE 4:**
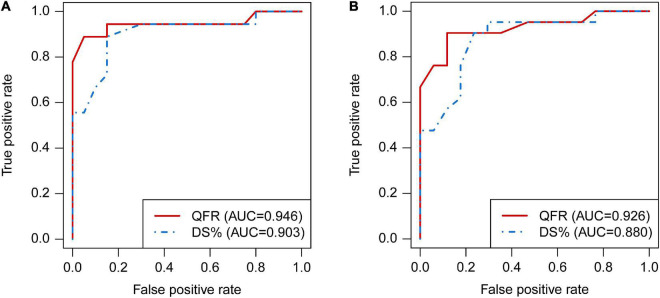
Comparison of diagnostic performance between QFR and DS% by receiver operating characteristic (ROC) analyses. The ROC curves of QFR and DS% in predicting FPR < 0.70 **(A)** and FPR < 0.75 **(B)**, respectively. QFR, quantitative flow ratio; DS, diameter stenosis; ROC, receiver operating characteristic; FPR, fractional pressure ratio.

When using FPR < 0.70 to define hemodynamically significance, the AUC of QFR was higher than DS% [0.946, 95% CI (0.820, 0.993%) vs. 0.903, 95% CI (0.762, 0.975%), *P* = 0.101], although this was not statistically significant (Figure 4). Similar results were obtained when setting FPR < 0.75 as the cut-off value [0.926, 95% CI (0.793, 0.985%) vs. 880, 95% CI (0.733, 0.962%), *P* = 0.147, Figure 4].

Table 3 demonstrates the diagnostic performance of dichotomous QFR and DS% in predicting hemodynamically significant lesions. According to the Youden index, the optimal cut-off values of DS% for predicting FPR < 0.70 and FPR < 0.75 were 50 and 45%, respectively. For predicting FPR < 0.70, the sensitivity of QFR < 0.70 was lower than DS% > 50% [88.9%, 95% CI (65.3, 98.6%) vs. 94.4%, 95% CI (72.7, 99.9%)], however, the specificity of QFR < 0.70 was higher than DS% > 50% [85.0%, 95% CI (62.1, 96.8%) vs. 70.0%, 95% CI (45.7, 88.1%)]. Diagnostic analyses for identifying FPR < 0.75 demonstrated similar results (Table 3).

**TABLE 3:** The diagnostic performance of dichotomous QFR and DS% in predicting hemodynamically significant lesions.

	Predicting FPR < 0.70		Predicting FPR < 0.75	
	QFR < 0.70	DS% ≥ 50%	QFR < 0.75	DS% ≥ 45%
AUC, % (95% CI)	0.869 (0.720, 0.956)	0.822 (0.664, 0.927)	0.870 (0.721, 0.957)	0.800 (0.643, 0.912)
Sensitivity, % (95% CI)	88.9 (65.3, 98.6)	94.4 (72.7, 99.9)	85.7 (63.7, 97.0)	95.2 (76.2, 99.9)
Specificity, % (95% CI)	85.0 (62.1, 96.8)	70.0 (45.7, 88.1)	88.2 (63.6, 98.5)	64.7 (38.3, 85.8)
PPV, % (95% CI)	84.2 (65.0, 93.9)	73.9 (59.0, 84.8)	90.0 (70.8, 97.1)	76.9 (63.5, 86.5)
NPV, % (95% CI)	89.5 (69.4, 97.0)	93.3 (67.1, 99.0)	83.3 (63.4, 93.5)	91.7 (61.1, 98.7)

*QFR, quantitative flow ratio; DS, diameter stenosis; FPR, fractional pressure ratio; AUC, area under the curve; CI, confidence interval; PPV, positive predictive value; NPV, negative predictive value.*

## Discussion

In this study, the novel computational QFR derived from a single angiographic view based on artificial intelligence algorithms achieved comparable results to the invasive FPR. Principal findings in this study are summarized as follows. First, excellent correlation and agreement can be seen between QFR and invasive FPR. Second, the computation of QFR shows high repeatability. Most importantly, QFR has excellent diagnostic performance in identifying hemodynamically significant stenosis.

The novel computational QFR in our study has comparable results to the invasive FPR. In previous studies, QFR computation was based on the three-dimensional (3D) geometry reconstruction of two angiographic views with an angle difference of at least 25° (Xu et al., 2017; Westra et al., 2019). Nevertheless, the novel QFR based on a single angiographic view achieved comparable diagnostic performance, which might be largely attributed to more accurate quantification of lesion severity. The new method for computing QFR took side branches into account, closer to the natural bifurcation physiology. A step-down reference diameter function based on the Murray bifurcation fractal law, instead of assuming linear tapering, was applied to reconstruct a more accurate reference vessel size (Tu et al., 2021). Although multiangle angiography can theoretically provide a more comprehensive description of lesion morphology, the suboptimal angiography view is often shortened and overlapped due to the curvature of cerebral arteries. Therefore, the suboptimal angiographic view may introduce additional errors, which further impair the accuracy of QFR.

Although the results were similar between QFR and FPR, some discrepancies were observed among certain patients. Several possible explanations might account for the deviation. First of all, the length of the vessel segment measured by the pressure wire was greater than that reconstructed in the QFR computation (Han et al., 2016). There might be a small consumption of flow reserve in the interrogated vessel due to non-significant stenoses. In theory, the extremely poor intravascular filling can also lead to bias in QFR computation. Furthermore, the inaccurate estimate of boundary conditions may also account for the differences. Nevertheless, slow frame rates of cerebral angiography and limited length of expansion lesions still hinder the calculation accuracy of individual flow velocity. Three-dimensional DSA images can be considered in future QFR computation. Notably, autoregulation and collateral circulation further complicate the cerebral hemodynamic status (Tian et al., 2021). More studies were required to continuously improve the method of QFR computation.

Our study validated the excellent diagnostic performance of QFR in identifying hemodynamically significant stenosis. Although, the diagnostic performance of QFR was better than that of DS% but did not reach statistical significance. Besides the small sample size, only the analysis of localized lesions may also contribute to the results. QFR was considered to have better prognostic performance than conventional angiographic assessment in tandem or multiple lesions (Rikuta et al., 2019). Hence, QFR has extensive application prospects and is worth more exploration.

Moreover, the automatic delineation of vessel contour based on an artificial intelligence algorithm facilitates rapid and stable QFR computation, enabling intraoperative immediate assessment. Although FFR is the gold standard for the functional evaluation of coronary stenosis, the inherent risks of invasive procedures and costs of guidewires limit its widespread use (Götberg et al., 2017; Tebaldi et al., 2018). Wire-free alternatives, such as CTA-derived FPR (Leng et al., 2015; Liu et al., 2017; Driessen et al., 2019; Zhuang et al., 2020) and MRA-derived FPR (Chen et al., 2020), had been proposed for the functional evaluation of cardio-cerebrovascular stenosis. Accumulating evidence demonstrated the prognostic value of image-based FPR for patients with ICAS (Leng et al., 2014, 2019), but the high time and technical requirements and labor intensiveness make the technique not accessible to routine clinical practice. Hence, the fast QFR computation has evident superiority in implementation in clinical practice, especially guiding revascularization strategies in the catheterization laboratory. Further studies are warranted to demonstrate the clinical value of QFR in the functional evaluation and risk stratification of patients with ICAS.

### Limitation

This study was subject to certain limitations. First, there was only a small validation set of FPR data available to assess the performance of QFR in this pilot study. Second, QFR computation can only be applied to fully exposed lesions rather than the entire vessel due to cerebrovascular tortuosity. In addition, potential changes in downstream vessel resistance induced by cerebral autoregulation also need to be taken into account.

## Conclusion

Computational QFR derived from a single angiographic view achieved comparable results to the wire-based FPR. The excellent diagnostic performance and repeatability empower QFR with high feasibility in the physiological assessment of cerebral arterial stenosis. Further studies are warranted to demonstrate the specific clinical significance of QFR for patients with cerebral artery stenosis.

## Data Availability Statement

The original contributions presented in the study are included in the article/supplementary material, further inquiries can be directed to the corresponding authors.

## Ethics Statement

This study was approved by the Ethics Committee of Jinling Hospital. The patients/participants provided their written informed consent to participate in this study.

## Author Contributions

KH, JD, and XL made contributions to conception and design. KH, WY, RL, YH, and WZ contributed to the acquisition of the data. KH, WY, YC, and ST contributed to analysis and interpretation of the data. KH, WY, RY, RL, and JD contributed to the drafting of the manuscript. JD, KH, ST, YC, RY, and XL were involved in critical revision of the manuscript for important intellectual content. All authors contributed to the article and approved the submitted version.

## Conflict of Interest

ST received research grants from Pulse Medical Imaging Technology. YC is a full-time employee of Pulse Medical Imaging Technology. The remaining authors declare that the research was conducted in the absence of any commercial or financial relationships that could be construed as a potential conflict of interest.

## Publisher’s Note

All claims expressed in this article are solely those of the authors and do not necessarily represent those of their affiliated organizations, or those of the publisher, the editors and the reviewers. Any product that may be evaluated in this article, or claim that may be made by its manufacturer, is not guaranteed or endorsed by the publisher.
